# Effect of Different Combinations of Freezing and Thawing Rates on the Shelf-Life and Oxidative Stability of Ostrich Moon Steaks (*M. Femorotibialis medius*) under Retail Display Conditions

**DOI:** 10.3390/foods9111624

**Published:** 2020-11-07

**Authors:** Coleen Leygonie, Louwrens Christiaan Hoffman

**Affiliations:** 1 Department of Food Science, University of Stellenbosch, Stellenbosch 7600, South Africa; cleygonie@gmail.com; 2 Department of Animal Sciences, Stellenbosch University, Private Bag X1, Matieland 7602, South Africa; 3 Centre for Nutrition and Food Sciences, Queensland Alliance for Agriculture and Food Innovation (QAAFI), The University of Queensland, Agricultural Mechanisation Building A, 8115, Office 110, Gatton 4343, Australia

**Keywords:** freezing rate, thawing rate, shelf-life, ostrich meat, oxidative stability, packaging

## Abstract

The aim of this study was to investigate the interaction between different rates of freezing and thawing on whole ostrich moon steaks to establish a combination or singular main effect that minimises thaw loss and maximises the retail display shelf-life regarding moisture loss, colour, lipid oxidation and tenderness. Five characteristic freezing rates (FR: 1, 2, 4, 8, 24 h) were compared with five characteristic thawing rates (TR: 1.5, 3, 6.5, 14, 21 h) in a completely randomised block design. Moon steaks (*M. femorotibialis medius*) from 125 birds were randomly assigned to a specific treatment combination before being subjected (after thawing) to a 10-day chilled storage at 4 °C shelf-life trial. Thawing rate had no effect (*p* > 0.05) on any of the quality (colour, drip and cooking losses, shear force, 2-thiobarbituric acid (TBARS)) parameters whilst freezing rate and display time both had significant (*p* < 0.05) influences. Thaw loss was lowest (*p* < 0.05) for the FR_1h and FR_2h, followed by FR_4h, FR_8h and FR_24. The FR_1h had the highest (*p* < 0.05) drip and shear force values during display while the FR_2h and FR_8h had the highest rate of oxidation (TBARS and metmyoglobin formation). FR_24h had the second best (*p* < 0.05) colour retention after FR_4h and minimal package drip. Overall, FR_4h resulted in the best quality meat over the entire shelf-life with high bloom retention, low TBARS and shear force, and average thaw, drip and cooking loss.

## 1. Introduction

South Africa is considered the world leader in the global ostrich marketplace with 90% of produced commodities (meat, leather and feathers) exported. With this high amount of exports, South Africa comprises 75% of the global market share with 45% skin, 45% meat and 10% feathers. Approximately 80,000 birds are slaughtered per annum in South Africa [1]. Approximately 90% of all the meat produced is exported, generally in a frozen state with subsequent thawing and processing upon arrival at the export market [2]. The majority of the South African processors freeze their export commodities in a convection blast freezer to a core temperature of −18 °C (takes >24 h to reach), at which temperature it is kept throughout transportation and storage. The mode of thawing is not regulated and is left to the client′s own discretion. This is regarded by the exporters as the site for quality losses but little research has been published quantifying the causes of quality loss [3]. Producers have recorded thaw losses in the range of 15–20% (calculated by weight loss) due to the freezing and thawing processes. The effect of the high thaw loss on the quality of the meat has not been studied in detail.

Freezing is an effective method of preservation against microbial spoilage [4]. However, a fraction of water (bound water) never completely freezes in the meat resulting in a degree of chemical reactivity during frozen storage that can negatively influence texture, colour and flavour. Recently, the effects of freezing rate, storage temperature and storage duration have been investigated extensively in the traditional species [4,5,6,7,8,9,10,11,12] and to a limited degree in ostriches [3,13,14] especially with regard to physiochemical changes and oxidative stability of the meat post-freezing and -thawing. The general conclusion is that an increase in freeze/thaw cycles or a reduction in the rate of freezing (characteristic freezing time increases) results in increased thaw and cooking losses reduced bloom and increased protein and lipid oxidation during retail display. The rate of deterioration that occurs in post-freeze/-thaw meat under retail display conditions is thus more rapid than for fresh meat in the traditional species [4].

The effect of thawing rate has a significant effect on the sensory and instrumental quality of the meat [15,16]. These authors did not study both freezing rate (FR) and thawing rate (TR) at the same time and hence an interaction could not be ruled out. In addition, most studies that reported on FR and/or TR were on smaller muscle samples and not on whole muscles. The latter is normally frozen before being transported by the various industries.

The aim of this study was to investigate the interaction between different rates of freezing and thawing on whole ostrich moon steaks (*M. femorotibialis medius*) to establish a combination or singular main effect that minimises thaw loss and maximises the retail display shelf-life regarding moisture loss, colour, lipid oxidation and tenderness.

## 2. Materials and Methods

### 2.1. Experimental Overview and Sample Preparation

#### 2.1.1. Experimental Layout

Consultation with the ostrich meat industry led to the selection of the moon steak (*M. femorotibialis medius*) for this study because it loses the most moisture during thawing. We obtained 250 moon steaks (*M. femorotibialis medius*) from a commercial abattoir in Oudtshoorn. They were collected from 125 birds (ca. 11–12 months old), selecting the left and the right muscle from each bird. The birds were randomly selected from the slaughter line to ensure unbiased selection. The muscles were collected on three slaughter occasions (50, 25, and 50 samples per occasion) to stagger the shelf-life trials so as to ensure homogeneity between batches, a specific treatment (FR_2h with TR_14h) was subjected to a shelf-life trial at each slaughter occasion so as to act as a standard/control. No differences were recorded between the controls of the three occasions, thereby indicating that the occasion of sampling had no influence on the main effects evaluated and could, therefore, be ignored in further statistical analyses.

An outline of the treatment combinations used in this study is shown in Table 1. Five different characteristic freezing rates were used. The characteristic freezing is the time it takes the thermal centre of the meat to transgress from 0 °C to −7 °C (FR) [17]. Each freezing treatment consisted of 25 muscles from 25 different birds; the freezing was ceased when an internal temperature of −20 °C was reached. The muscles were then transferred to a holding freezer (−20 °C) and stored for 30 days where after the 25 muscles were randomly assigned to five different characteristic thawing rates (TR; time it takes to transgress from −7 °C to 0 °C). Each freeze-thaw combination thus had five replications.

The freezing and thawing rates were measured by inserting a thermocouple into the thermal centre of three muscles per treatment. The thermocouples (Tipped probe, ST100T-15, LogTag, Auckland, New Zealand) were connected to thermo data loggers (Trex-8, LogTag, Auckland, New Zealand) and set to measure the temperature every minute. 

#### 2.1.2. Freezing

The different freezing rates were achieved by changing the combination of freezing medium and insulation; all muscles were vacuum-packed (oxygen transmission rate 38 cm^3^/m^2^/24 h; water vapour transmission rate 3.0 g/m^2^/24 h; carbon dioxide transmission rate 205 cm^3^/m^2^/24 h). The FR_1h was achieved by immersing the vacuum-packed muscles into a brine freezer (27% NaCl) set at −20 °C and the FR_2h in a blast freezer set at −25 °C with an average wind speed of 2.6 m/s. For the FR_4h and FR_8h, respectively, one and two layers of newspaper were placed around the individual vacuum packed muscles and frozen in the blast freezer set at −25 °C with an average wind speed of 2.6 m/s. The FR_24h depicting a typical commercial rate, was achieved by wrapping the vacuum packed muscle in a polystyrene box (0.8 mm diameter) and freezing in a convection freezer set at −20 °C. For all treatments, freezing was considered complete once an internal temperature of −12 °C was reached. The samples were then transferred to a holding freezer to equilibrate further to −20 °C.

#### 2.1.3. Thawing

After being frozen at −20 °C for four weeks, the muscles were removed for the thawing treatments. The five methods of thawing were achieved using two thawing mediums and various temperatures. Characteristic thawing rates (TR) of 1.5 h and 3 h were achieved by placing the vacuum-packaged frozen muscles in a water bath set to 10 °C and 5 °C, respectively. Allowing the samples to thaw in a convection fridge with minimal air flow at 10 °C gave rise to TR_6.5h, at 4 °C with no added insulation TR_14h, and with insulation (two layers of newspaper) TR_21h. Thawing was considered complete once the internal temperature reached 4 °C.

#### 2.1.4. Shelf-Life

Each thawed muscle was cut into six 1 cm thick steaks (weighing approximately 85 g each) which were randomly allocated to the sampling days (0, 2, 4, 6, 8, and 10). The steaks were packaged in polystyrene trays and covered with Versafilm (Crown National, Montague Gardens, Cape Town, South Africa) with a moisture vapour transfer rate of 585 g/m^2^/24 h/1 atm, O_2_ permeability 25,000 cm^3^/m^2^/24 h/1 atm and a CO_2_ permeability of 180,000 cm^3^/m^2^/24 h/1 atm. Care was taken that the film did not touch the surface area of the steak. The samples were stored for 10 days at 4 °C with illumination of 870 lux, and samples were analysed every 2 days from day 0 (taken as the day the samples were thawed and packaged).

### 2.2. Physico-Chemical Parameters

#### 2.2.1. Moisture Losses

Each muscle was weighed before packaging and freezing. After thawing the muscles were removed from the packaging, blotted dry with paper toweling, and weighed. The thaw loss was calculated as a percentage difference between the initial pre-freezing weight and the post-thawing/drying weight. 

Prior to packaging for the shelf-life investigation, each steak was weighed. On the designated sampling day, the steaks were blotted dry with tissue paper and re-weighed. The difference was calculated and expressed as a percentage drip loss calculated from the initial weight of the sample. 

On each sampling day weighed steaks (1 cm thick, weighing ±85 g) from each treatment combination were cooked in polyethylene bags in a water bath (±80 °C) for 60 min, after which the water was drained from the bags and the samples allowed to cool (±5 °C, still in plastic bags) before being patted dry with tissue paper and weighed. Cooking loss was calculated as the percentage of weight lost by each sample.

#### 2.2.2. pH

The pH of the centre of each raw steak was measured on each sampling day, using a Testo 205 pH (Testo AG, Lenzkirch, Germany) glass meat probe that was inserted into the steak perpendicular to the muscle fibres.

#### 2.2.3. Surface Colour

##### CIE L*a*b* and Oxymyoglobin: Metmyoglobin ratio

The surface colour (CIE L*a*b*) of the raw ostrich steaks was measured using a Color-guide D65/10° (daylight illumination, aperture opening) 45°/0° colorimeter (BYK-Gardner GmbH, Gerestried, Germany). Five measurements were taken on each steak immediately upon opening the package. The Hue angle (h_ab_) (°) and Chroma (C*) were also calculated from the individual CIE a* and b* values. The average of the five readings was used in the statistical analysis. The oxymyoglobin to metmyoglobin ratio was calculated as the absorbance at 580/630 nm.

#### 2.2.4. Lipid Oxidation

Lipid oxidation was assessed by the 2-thiobarbituric acid (TBARS) extraction method. Core samples (1.0 × 1.0 × 1.0 cm meat block from the centre of the steak) from each treatment combination steak were collected and analysed the same day. Analysis was conducted on 1 g of core sample and the TBARS concentrations were calculated using 1,1,3,3-tetramethoxypropane (0–20 μM) as a standard and expressed as milligram malonaldehyde (MDA) per kilogram of meat.

#### 2.2.5. Shear Force (Warner–Bratzler)

The cooled (±4 °C) cooking-loss samples were used for the Warner-Bratzler shear force test. Three 12.7 mm diameter cylindrical core samples were cut parallel to the muscle fibre direction of each cooked sample at randomly chosen sites taking care to avoid visible connective tissue. The average force (Newton) required to shear through the core samples was measured with an Instron Universal Testing Machine (Model 4444, Apollo Scientific, Johannesburg, South Africa) fitted with a Warner Bratzler blade, 1.2 mm thick with a triangular opening (13 mm at the widest point and 15 mm high), fitted with a 2 kN load cell and set to a crosshead speed of 100 mm/min.

### 2.3. Statistical Analysis

Univariate analysis of variance (ANOVA) was performed on all variables for each day of the shelf-life trial as well as a split-plot ANOVA with day as a sub-plot factor, using the GLM (General Linear Models) Procedure of SAS statistical software version 9.1 [18]. In both cases, freezing rate and thawing rate were main effects including all possible higher order interactions. A Shapiro-Wilk test was performed to test for normality in all results. Student’s *t*-least significant differences were calculated at the 5% level to compare treatment means. A probability level of 5% was considered significant for all significance tests. In addition the data was subjected to multivariate methods namely principal component analysis (PCA), Discriminant analysis (DA) and Pearson correlation tests using XLStat, Version 2007.8.03 (Addinsoft, New York, NY, USA).

## 3. Results and Discussion

**Figure 1:**
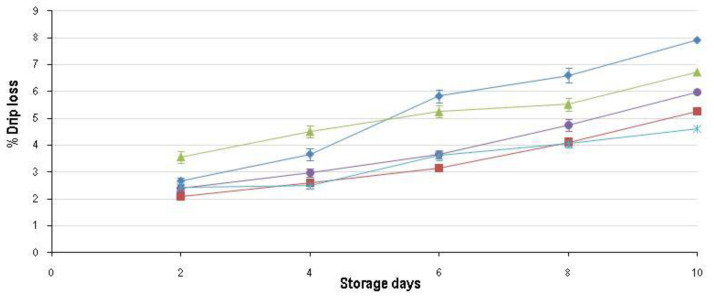
Average drip loss (±std. error) for the five characteristic freezing rates: FR_1h (♦); FR_2h (■); FR_4h (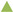); FR_8h (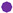) and FR_24h (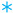) over the 10-day shelf-life trial at ±4 °C.

**Figure 2:**
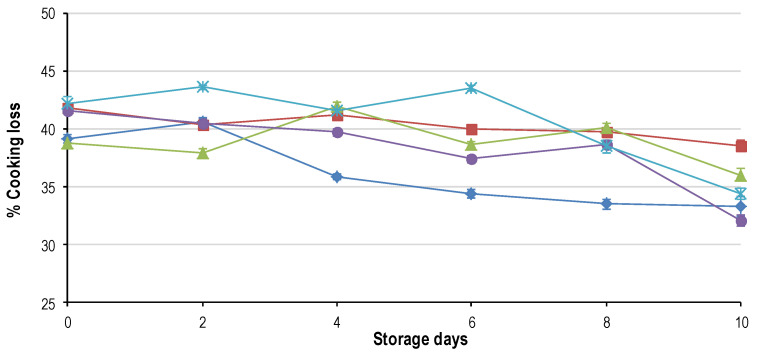
Average cooking loss (±std. error) for the five characteristic freezing rates: FR_1h (♦); FR_2h (■); FR_4h (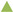); FR_8h (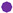) and FR_24h (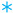) over the 10-day shelf-life trial at ±4 °C.

**Figure 3:**
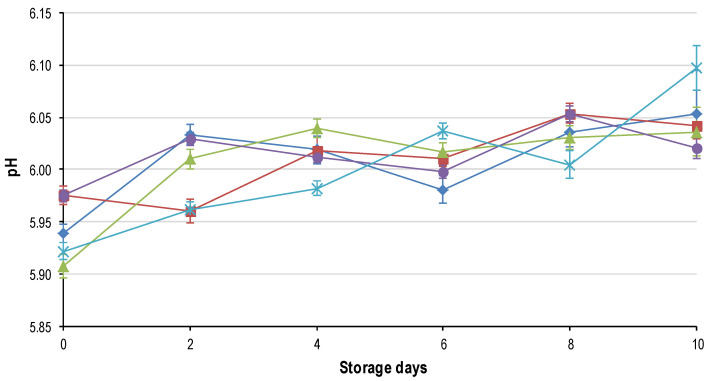
Average pH (±std. error) for the five characteristic freezing rates: FR_1h (♦); FR_2h (■); FR_4h (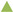); FR_8h (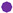) and FR_24h (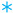) over the 10-day shelf-life trial at ±4 °C.

**Figure 4:**
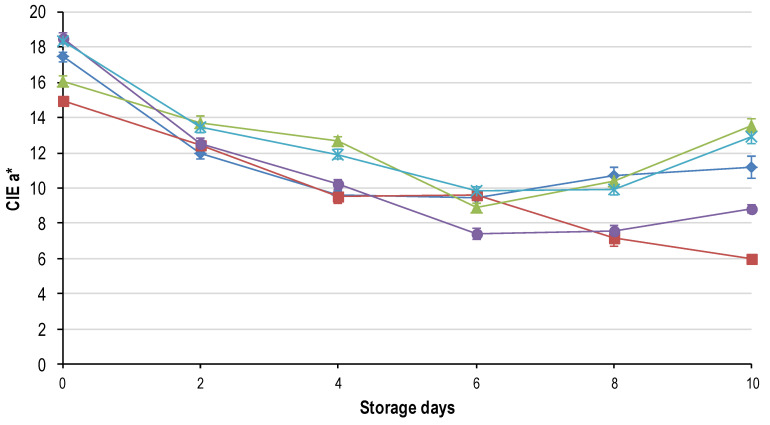
Average CIE a* (±std. error) for the five characteristic freezing: FR_1h (♦); FR_2h (■); FR_4h (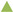); FR_8h (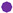) and FR_24h (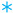) over the 10-day shelf-life trial at ±4 °C.

**Figure 5:**
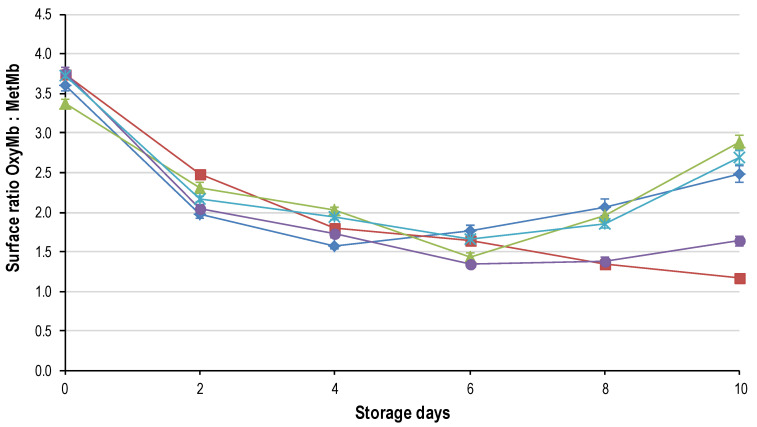
Ratio oxymyoglobin (OMb)/metmyoglobin (MMb) (±std. error) for the five characteristic freezing rates: FR_1h (♦); FR_2h (■); FR_4h (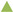); FR_8h (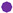) and FR_24h (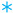) over the 10-day shelf-life trial at ±4 °C.

**Figure 6:**
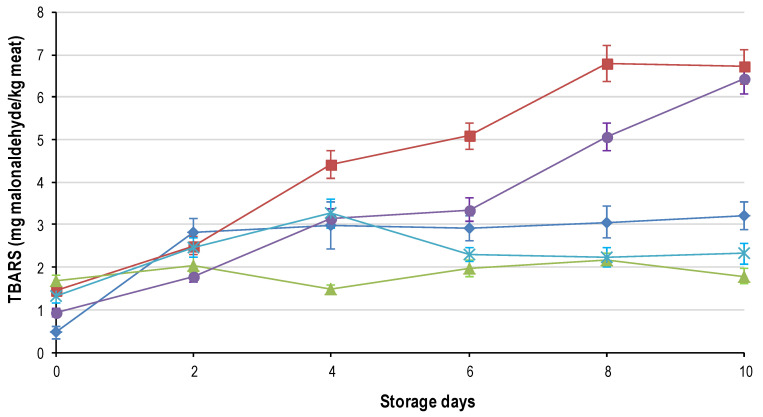
Average 2-thiobarbituric acid (TBARS, ±std. error) for the five characteristic freezing rates: FR_1h (♦); FR_2h (■); FR_4h (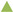); FR_8h (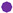) and FR_24h (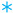) over the 10-day shelf-life trial at ±4 °C.

**Figure 7:**
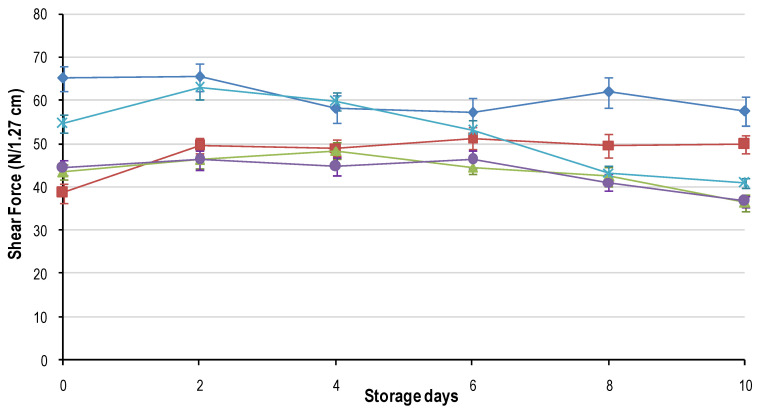
Average shear force (N/1.27 cm) (± std. error) for the five characteristic freezing rates: FR_1h (♦); FR_2h (■); FR_4h (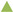); FR_8h (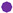) and FR_24h (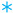) over the 10-day shelf-life trial at ±4 °C.

**Figure 8:**
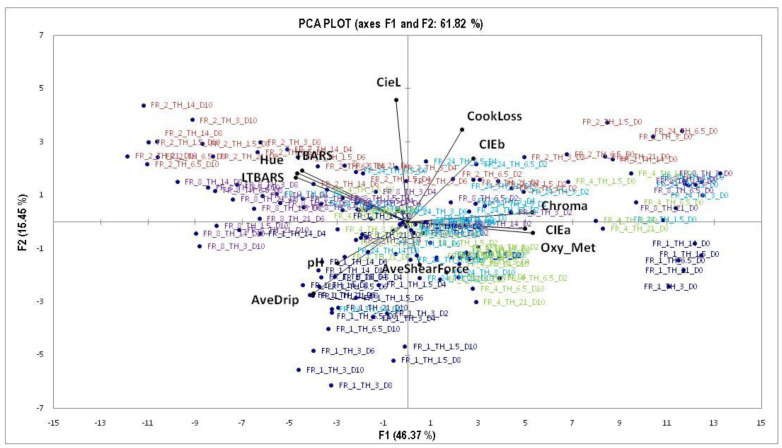
Principle component analysis (PCA) of quality parameters and the combined freezing and thawing treatments over the 10-day shelf-life trial at ±4 °C. The freezing treatments have been colour coded for the five characteristic freezing rates: FR_1h (♦); FR_2h (■); FR_4h (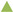); FR_8h (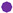) and FR_24h (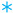).

The ANOVA was performed for each sampling day (per day) and revealed that only the main effect of freeze rate (termed freeze) was significant for all the quality parameters on each day. The main effect of thaw rate (termed thaw) had no effect on the final quality of the meat on any of the sampling days. The split plot ANOVA, with day as a sub-plot and freeze and thaw rate as main effects, indicated that only the freezing rate had a significant effect on the quality (as defined by [19]) of the final product. In addition, the split-plot ANOVA showed that there was a significant interaction between the main effects of freeze rate and day for all the quality parameters. This indicates that only freezing rate and display time had an effect on the deterioration of quality. The results from these analyses are presented in Figure 1, Figure 2, Figure 3, Figure 4, Figure 5, Figure 6, Figure 7 and Figure 8.

### 3.1. Moisture Loss

The loss of moisture upon thawing (percentage thaw loss) was subjected to analysis of variance, which revealed that only the main effect freeze was significant and that the thawing regime had no effect (*p* > 0.05). The degree of thaw loss increased as the rate of freezing increased, except for FR_1h and FR_2h that did not differ (*p* > 0.05) from each other, all the other treatments differed significantly (Table 2).

The rate of thawing had no effect on the amount of moisture lost during thawing. This is contradictory to earlier work reporting differences in losses between thawing (from −18 °C to 0 °C) rates ranging from 28 h, 5–7 h, 1.5 h and 35 min [15]. Decreases in thaw loss as the rate of thawing decreased (characteristic thawing time increased) were also noted in beef [20] and in ostrich [16]. In the present investigation, the meat was stored (−20 °C) for 4 weeks and a significant difference between different freezing rates was still evident (Table 2). This is contradictory to results noted in pork where, as the duration of frozen storage increased to 4 weeks, the effect of the freezing rate disappeared [21]. The increase in thaw loss with an increase in the rate of freezing, however, does correspond to the “no storage duration” findings in pork [21]. As the rate of freezing became slower the size and location (from intracellular (i.e., FR_1h) to extracellular) of the ice crystals changes which causes increased damage to the ultrastructure of the meat and results in more thaw loss [4] due to incomplete reabsorption of the extracellular water [20]. The size and location of the ice crystals are, thus, believed to have played a significant role in the moisture lost during thawing.

Moisture loss is a major concern in dealing with frozen meat; the results in Table 2 indicate that thaw loss (average of all the thaw treatments as thaw rate had no effect) increased significantly as freezing rate increased. However, thaw losses are not the only moisture loss concern. Drip loss (moisture loss during display) of packaged meat is also important, especially since it plays a prominent role in the aesthetic appeal to the consumer. The percentage drip loss (Figure 1) was mainly influenced by freezing rate and secondly by the thawing rate (only significant on days 2 and 4). On days 2 and 4, FR_4h lost significantly more fluid than all other treatments; from day 6 through 10, FR_1h lost significantly more moisture than all other treatments. These freezing rates lost more fluid than the rest for different hypothesized reasons. FR_4h lost more moisture due to the damage to the ultrastructure because of the ice crystal formation [4], which resulted in the inability of the myofibrils to contain the moisture [20,22]. For FR_1h, a large amount of intracellular ice crystals formed resulting in a low thaw loss (Table 1) but more severe protein damage [3]. The rapid increase in drip loss (Figure 1) could thus be attributed to the decrease in ability of the water to interact with the proteins. The decrease in interaction thus led to accelerated movement of ‘free’ and ‘entrapped’ water from the intracellular spaces to the extracellular space. The water then moved out of the meat through the enlarged ‘drip loss channels’ that formed during the shrinkage of the myosin lattice post mortem [23].

Overall, for drip loss over the shelf-life FR_1h showed the most rapid increase, followed by FR_4h and FR_8h, FR_2h and finally FR_24h that lost the least amount. Therefore, except for FR_2h, increased thaw loss corresponds to decreased drip loss. Meat only has a certain amount of free and entrapped moisture [23] to release by these different mechanisms. Therefore, if a large percentage of the moisture has already been lost during thawing (e.g., FR_24h ± 6% thaw loss) less water can be lost during display. FR_2h likely had the correct balance between intra- and extracellular ice crystals to cause minimal damage and destruction to the muscle fibre matrix and proteins [22], and hence the capillary forces that hold the water within the meat remained intact.

Even though freezing rate played a more significant role in drip loss, thawing rate initially (days 2 and 4) had a significant effect irrespective of freezing treatment (Table 1). On both these days, the samples thawed in water (TR_1.5h and TR_3h) lost significantly less moisture than the samples thawed in air. This advantage, however, faded over time. As the meat was vacuum packaged with minimal moisture and air permeability the likely cause was related to the rate of thawing and not the medium in which the meat was thawed.

The freeze and storage day interaction (split-plot ANOVA) was likely significant due to FR_1h crossing over FR_4h between day 4 and 6 (Figure 1). The main effect ′day′ was also significant, which is in accordance with others [24,25].

Only the freezing rate had a significant effect on the cooking loss on each of the storage days. Cooking loss and drip loss were negatively correlated (R^2^ = 0.402, *p* < 0.0001) indicating that as the drip loss increased (Figure 1) the cooking loss decreased (Figure 2). It seems that the same ice crystal damage that caused the slow frozen samples (FR_24h) to lose more moisture during thawing also caused higher cooking losses. Initially, FR_2h, FR_8h and FR_24h lost significantly more moisture than FR_4h and FR_1h. Over time, FR_2h did not change; FR_4h and FR_8h remained relatively constant but started decreasing from day 8 onwards. FR_24h was significantly higher for days 0 through 6 where after a rapid decrease commenced and FR_1h decreased rapidly over the entire shelf-life.

The increasing temperature during cooking first causes the myofibrillar proteins to denature leading to a loss in water holding capacity [26]. Then the proteins start to coagulate causing shrinkage of the myofilament lattice resulting in more fluid loss and toughening. Preliminary work indicated that freeze/thaw treatment did not affect cooking loss [3]. However, the results from this study clearly show how time and freezing rate affect cooking loss; this could be attributed to the fact that only one rate of freezing was used in the earlier study [3] and thus the difference was not as pronounced. If Warriss’ [26] reasoning is followed, the degree of damage caused by the ice crystals shows a connection to cooking loss. Minimal damage (FR_1h and FR_2h) resists compression due to intact muscle fibre matrixes. The reason for the decrease in FR_1h over time compared to no change in FR_2h is therefore due to the high drip loss in FR_1h. The other treatments all suffered great disruption of the muscle fibre matrix due to ice crystal formation, leading to increased compression of the myofilament lattice during cooking and thus more moisture being expelled. The decrease in cooking loss over time also goes to show that the reasoning that meat contains a fixed amount of moisture holds true. As this moisture is lost to thaw and drip loss less remains to be lost during cooking.

In summary, for moisture loss as a whole it seems that the rate of freezing directs the differential loss of moisture (i.e., thawing, display or cooking). FR_1h had the lowest thaw loss, highest rate of drip loss and lowest cooking loss. FR_2h had the same thaw loss as FR_1h, the slowest rate of drip loss and constant cooking loss. FR_4h had a medium amount of thaw loss, second highest rate of drip loss and a relatively constant cooking loss. FR_8h had the second largest thaw loss, third highest drip loss and decreasing cooking loss from day 6. FR_24h had the greatest thaw loss, the least drip loss and initially the highest cooking loss that decreased from day 6 onwards.

### 3.2. pH

The pH increased (*p* < 0.05) gradually over time (Figure 3) in all the freezing treatments. Even though the freezing rate had a significant effect on days 0 through 8, the split-plot ANOVA showed a very weak significant interaction for freeze*day and freeze. However, a strong significance (*p* < 0.0001) for the main effect of day was evident. This suggests that the increase in pH was most likely more affected by time than by the freezing rates.

The initial pH of all the samples was approximately 5.95, which is lower than reported for fresh ostrich (±6.08) [3,27]. This would seem to indicate that freezing leads to a decrease in the pH of ostrich meat. However the influences of different batches of birds, slaughter environments and a number of other factors need to be taken into account before this can be validated or the mechanism elucidated. The increase in pH over time is customary for ostrich meat [13,14]. The reason for the increase is thought to be partly connected with microbial spoilage (predominantly *Pseudomonas*) and the change in metabolism from glucose to amino acids [28]. The deamination of the amino acids results in the formation of ammonia (NH_3_) formed by the addition of an H^+^ to the NH_2_^−^ group cleaved from the amino acid. The uptake of H^+^ results in a pH increase [27].

### 3.3. Surface Colour

The colour of the meat was evaluated with two methods namely, CIE L*a*b* with calculated hue and chroma and the ratio of oxymyoglobin (OMb) to metmyoglobin (MMb) on the surface. Freezing rate had an effect on each sampling day for all the colour parameters, which is contradictory to literature reports [29,30,31] where no differences were found. The CIE L* (lightness) did not change dramatically during the shelf-life trial oscillating from 30–35 in all the treatments. The CIE b* (yellowness) decreased gradually over time for all the freeze treatments from approximately 10.31 ± 0.33 (day 0) to 9.15 ± 0.15 (day 10).

The redness (CIE a*; Figure 4) was initially significantly greater in the slow frozen (FR_24h and FR_8h) samples, followed by FR_1h, Fr_4h and FR_2h. This differs slightly from the OMb:MMb (Figure 5) in that all the treatments had the same ratio of OMb:MMb except for FR_2h, which was significantly lower. The difference between the OMb:MMb and the CIE a* is that the former gives a better illustration of the consumer’s colour perception [29]. However, the CIE a* correlated well with OMb:MMb (*p* < 0.0001; R^2^ = 0.933). The initial values for all freezing rates were lower than reported for fresh ostrich steaks (CIE a* 19.62 ± 0.45) [3] an important observation as consumers relate colour to the freshness of the meat [29].

The decline in redness (CIE a* and OMb:MMb) over time (Figure 4 and Figure 5) was noticeable from day 0–6, where after a slight increase was evident in most of the treatments. The increase from day 8 to 10 can be attributed to microbial spoilage and the formation of a slime layer on the surface. The slime layer altered the reflectance spectra seeming to increase the redness of the meat [28]. The rapid drop-off in redness between day 0 and 2 can be attributed to the freeze/thaw treatment. During frozen storage the metmyoglobin reducing activity (MRA) governed by the NADH-cytochrome b_5_ reductase enzyme is lost causing more rapid accumulation of metmyoglobin on the surface of the meat [29]. In conjunction, freezing causes the release of the mitochondrial enzyme β-hydroxyacyl CoA-dehydrogenase (HADH). This enzyme utilizes NADH thereby depleting the co-factors used by the MRA and further accelerating the formation of metmyoglobin [29]. This is supported by the increase in hue angle over time (an indication of metmyoglobin accumulation), which was negatively correlated (*p* < 0.0001; R^2^ = 0.660) to OMb:MMb (Figure 5).

The significant difference achieved between freezing treatments for colour was not demonstrated by others [30,31]. It is argued that the degree of damage to the ultra-structure of the meat increases with a decrease in freezing rate and it would then be expected that the most rapidly frozen samples would have better colour stability. The results contradicted this in that the slow frozen (FR_24h) and the medium rate of freezing (FR_4h) had the best colour stability (Figure 4 and Figure 5). The FR_2h and FR_8h deteriorated at the fastest rates with FR_1h narrowly behind. A positive correlation between lipid oxidation (TBARS) and the CIE a* (*p* < 0.001; R^2^ = 0.518) suggests that lipid and myoglobin oxidation is interconnected [32] in ostrich meat, and thus the rapid colour deterioration is likely a result of the rapid lipid oxidation (Figure 6). However, there is still much debate regarding whether lipid or myoglobin oxidation is the initiator of the chain reaction of oxidation. At this point, we can only say that the two systems did have an effect on each other in this study. FR_2h and FR_8h were more susceptible to oxidation than the other treatments, likely because of the following: cell membrane puncturing (exposing phospholipids), heme iron exposure or enzyme activation due to the size, location and degree of damage caused by the ice crystals.

### 3.4. Lipid Oxidation (2-Thiobarbituric Acid (TBARS))

Lipid oxidation (TBARS, Figure 6) and colour deterioration were significantly correlated (CIE a* −0.720, R^2^ 0.518; OMb:MMb −0.638, R^2^ = 0.408), indicating that as metmyoglobin accumulated on the surface of the meat, the TBARS increased. This strong correlation provides further evidence that the lipid and myoglobin oxidation reactions are interdependent [32]. Lipid oxidation was significantly affected (on each sampling day) by the freezing rate but not the thawing rate. This is contradictory to Hong [6] who reported that the thawing rate had a significant effect on the quality of ostrich meat. All TBARS values of the freeze treatments increased (*p* < 0.05) from day 0 to day 2, with FR_1h changing the most from the lowest value on day 0 to the highest on day 2. From days 2 through 10, FR_2h and FR_8h increased rapidly while the rest of the freeze treatments reached a plateau. FR_4h showed the lowest overall TBARS and the least amount of change over the entire shelf-life trial.

Only storage duration and not freezing rate was found to have a significant effect on lipid oxidation (TBARS) of chicken and salmon muscle [33]. Many authors [12,34] have since shown that frozen storage duration overshadows the effect of freezing rate; a modest one-month’s storage at −20 °C was sufficient to mitigate all advantages of rapid freezing. This is contradictory to the results obtained in this study where the meat had been frozen for one month. However, in lamb it was reported that as the length of frozen storage increased the freezing rate effect became more significant [11]. This demonstrates that there are still major conflicting results in literature regarding the effect of freezing rate on lipid oxidation.

This study clearly showed that freezing rate had a significant effect on the rate of lipid oxidation during display post-freeze/-thaw. The reason for the higher rate of oxidation in FR_2h and FR_8h was previously alluded to. However there is increasing evidence that lipid oxidation takes place primarily at the cell membrane level (Thanonkaew et al., 2006) and hence ice crystal formation could rupture the cell membranes [35] exposing pro-oxidants and increasing the reactive area thereby causing rapid TBARS accumulation. These present results can thus be explained as follows; FR_1h had mainly intracellular ice crystals with membrane rupture unlikely, although HADH release is likely responsible for the increased rate of discolouration. The slower freezing rate (FR_24h) may have resulted in columns of ice forming which might have smoother edges and thus minimal cell membrane damage occurring even though the entire muscle fibre matrix was distorted. FR_4h most probably had major disruption of the muscle fibre matrix but the degree of muscle fibre shrinkage was not as pronounced as FR_2h and FR_8h, indicating that cell membrane permeability was likely compromised. More research is required into the shape of the crystals that form at the various freezing rates (as postulated) and the integrity of muscle fibre membranes to determine how these affect the meat quality attributes.

### 3.5. Toughness (Shear Force)

The toughness (Figure 7) of the meat was only influenced by freeze treatment. Over all the storage days, FR_1h consistently had the toughest meat (65–57 N). FR_24h followed suit on days 0 through 4 (peak at 63.02 ± 2.54), where the toughness rapidly decreased to reach a minimum on day 10 (41.02 ± 1.17). The other freeze treatments increased slightly from day 0 to day 2 and remained constant up to day 6 where after FR_4h, FR_8h decreased rapidly, and FR_2h continued to remain constant. Therefore, if a baseline value for shear force for fresh meat is set at 58–110 N as stated in [36], then literaturefreezing at rates between FR_2h and FR_8h could be seen to have a tenderizing effect. This is likely due to the structural damage caused by the ice crystals as postulated. For FR_1h the values reported in this study are above those for the baseline, which is contradictory to other researchers [36,37] who reported that a rapid rate of freezing resulted in more tender meat. It is argued that minimal structural damage occurred in FR_1h due to the dominance of intracellular ice crystals, and therefore the integrity of the muscle fibres were retained resulting in no tenderizing effect by freezing. As for FR_24h, the initial high shear force values could be attributed to the high loss in moisture, which caused compression of the muscle fibres resulting in more muscle tissue per area and higher peak forces. However, severe ultrastructure damage as caused by the ice crystals and changes in the proteins because of the freeze/thaw cycle likely resulted in the proteins being more accessible to the tenderizing enzymes resulting in tenderization over time.

The question that remains unanswered is whether the excessive loss of moisture in FR_4h, FR_8h and FR_24h will counteract the decrease in shear force and negatively influence the sensory palatability of the meat. This warrants further study into the sensory effect of various freezing rates.

### 3.6. Principle Component Analysis (PCA)

The first two principle components (F1 and F2) explained 61.82% of the overall variation in the meat quality data (Figure 8). From the discriminate analysis (DA, results not shown), thawing rate had no significant effect on meat quality (as defined by [20]) as no clear separation could be seen between the five treatments. The DA for freezing rate showed partial separation of the five rates, which has been highlighted in the PCA plot (Figure 7).

Factor 1 (F1) declares the largest amount of variation possibly due to the effect of storage time as it moved from predominantly day 0 on the right to day 10 on the left. Additionally, the freezing treatments in and of themselves also grouped from right to left. FR_2h and FR_8h correlated with high hue and TBARS values, both negative characteristics and indicative of high susceptibility to oxidation. This could result in unacceptable quality cues to the consumer because of an unattractive colour and rancid flavour. FR_1h was highly correlated with high drip loss and shear forces, which indicate that although the meat is not as susceptible to oxidation, it loses high amounts of moisture during display and is the toughest of all the freezing treatments. This could result in the meat being discriminated against by the consumer due to toughness and lack of juiciness. FR_24h is positioned centrally between PCA 1 and PCA 2, indicating that neither of the quality traits significantly correlated with observations and no clear conclusion could be reached. FR_4h was positioned in the most promising area, as it correlates well with the redness indices (CIE a*, OMb:Mmb and Chroma) indicating a sustained bloomed colour, low oxidation susceptibility and reasonably low cooking loss, the only negative is the close proximity to drip loss. Nevertheless, FR_4h has been shown to provide the retailer with a prolonged bloomed state during display to attract the customer, and the consumer should be satisfied by the sensory experience, as the meat will be tender and lose minimal moisture loss during cooking.

An aspect that warrants further research is to quantify the effects of these or similar freezing and thawing rates on the microbiological as well as sensory attributes of the resultant meat when stored under various conditions such as MAP and/or oxygen permeable packaging.

## 4. Conclusions

The aim of this study was to find a combination of freezing and thawing rates that would result in the best quality ostrich meat. Thawing rate was found to have no significant influence on any of the quality parameters tested. Hence, only the freezing rate had an effect on meat quality, and the best overall quality ostrich meat for retail conditions display was FR_4h. FR_4h was the intermediate rate of freezing even though FR_1h resulted in the lowest thaw loss, over all, FR_4h outperformed all the other rates of freezing to yield a superior product. The commercial rate of freezing (FR_24h) resulted in the largest thaw loss but on all other quality parameters it faired reasonably well to yield a product of moderate quality.

The most rapidly frozen meat (FR_1h) lost minimal moisture during thawing but the meat was extremely tough and had high package weep which hindered the attractiveness of the meat under display as well as the sensory experience the consumer would have. The remaining freezing rates evaluated, FR_2h and FR_8h produced the poorest quality meat. These freezing rates resulted in meat that was highly susceptible to oxidation resulting in meat that could be perceived by consumers to be unattractive both visually (high levels of metmyoglobin) and organoleptically (high TBARS). More research is required to establish whether consumer preference and instrumental quality assessments coincide with the effect of different rates of freezing.

## Figures and Tables

**Table 1:** Characteristic freezing (FR) and thawing rate (TR) combinations applied to ostrich moon steaks (*n* = 5 birds per combination).

Treatment	TR_1.5h *	TR_3h	TR_6.5h	TR_14h	TR_21h	Sample Number Per Treatment
FR_1h*	*n* = 5 birds	*n* = 5 birds	*n* = 5 birds	*n* = 5 birds	*n* = 5 birds	*n*_FR_1h_ = 25
**FR_2h**	*n* = 5 birds	*n* = 5 birds	*n* = 5 birds	*n* = 5 birds	*n* = 5 birds	*n*_FR_2h_ = 25
**FR_4h**	*n* = 5 birds	*n* = 5 birds	*n* = 5 birds	*n* = 5 birds	*n* = 5 birds	*n*_FR_4h_ = 25
**FR_8h**	*n* = 5 birds	*n* = 5 birds	*n* = 5 birds	*n* = 5 birds	*n* = 5 birds	*n*_FR_8h_ = 25
**FR_24h**	*n* = 5 birds	*n* = 5 birds	*n* = 5 birds	*n* = 5 birds	*n* = 5 birds	*n*_FR_24h_ = 25
**Sample number per treatment**	*n*_TR_1.5h_ = 25	*n*_TR_3h_ = 25	*n*_TR_6.5h_ = 25	*n*_TR_14h_ = 25	*n*_TR_24h_ = 25	*n*_total_ = 125

* indicates the duration of the freezing or thawing in hours.

**Table 2:** Average percentage thaw loss for all the freezing treatments (± SE) calculated for the intact whole muscle.

Freeze Treatment	Average Thaw Loss (%) ± SE
FR_1h	2.57 ^d^ ± 0.35
FR_2h	3.00 ^d^ ± 0.21
FR_4h	3.93 ^c^ ± 0.35
FR_8h	5.26 ^b^ ± 0.30
FR_24h	6.24 ^a^ ± 0.32

^a–d^ letters in the same column indicate a significant difference at the 5% level.

## References

[1] Barends-Jones V., Pienaar L. (2020). The South African Ostrich Industry Footprint.

[2] Department of Agriculture, Forestry & Fisheries (DAFF) (2017). A Profile of the South African Ostrich Market Value Chain.

[3] Leygonie C., Britz T.J., Hoffman L.C. (2012). Meat quality comparison between fresh and frozen/thawed ostrich *M. iliofibularis*. Meat Sci..

[4] Leygonie C., Britz T.J., Hoffman L.C. (2012). Impact of freezing and thawing on the quality of meat: Review. Meat Sci..

[5] Kim H.W., Kim Y.H.B. (2017). Effects of aging and freezing/thawing sequence on quality attributes of bovine *Mm. gluteus medius* and *biceps femoris*. Asian-Australas. J. Anim. Sci..

[6] Kim H.W., Kim J.H., Seo J.K., Setyabrata D., Kim Y.H.B. (2018). Effects of aging/freezing sequence and freezing rate on meat quality and oxidative stability of pork loins. Meat Sci..

[7] Kim Y.H.B., Liesse C., Kemp R., Balan P. (2015). Evaluation of combined effects of ageing period and freezing rate on quality attributes of beef loins. Meat Sci..

[8] Ali S., Zhang W., Rajput N., Khan M.A., Li C.B., Zhou G.H. (2015). Effect of multiple freeze–thaw cycles on the quality of chicken breast meat. Food Chem..

[9] Vieira C., Diaz M.Y., Martínez B., García-Cachán M.D. (2009). Effect of frozen storage conditions (temperature and length of storage) on microbial and sensory quality of rustic crossbred beef at different stages of aging. Meat Sci..

[10] Xia X., Kong B., Lui Q., Lui J. (2009). Physiochemical change and protein oxidation in porcine *longissimus dorsi* as influenced by different freeze/thaw cycles. Meat Sci..

[11] Muela E., Sañudo C., Campo M.M., Medel I., Beltrán J.A. (2020). Effect of freezing method and frozen storage duration on instrumental quality of lamb throughout display. Meat Sci..

[12] Soyer A., Özalp B., Dalmış Ü., Bilgin V. (2010). Effects of freezing temperature and duration of frozen storage on lipid and protein oxidation in chicken meat. Food Chem..

[13] Leygonie C., Britz T.J., Hoffman L.C. (2011). Oxidative stability of previously frozen ostrich *Muscularis iliofibularis* packaged under different modified atmospheric conditions. Int. J. Food Sci. Technol..

[14] Leygonie C., Britz T.J., Hoffman L.C. (2011). Protein and lipid oxidative stability of fresh ostrich *M. iliofibularis* packaged under different modified atmospheric packaging conditions. Food Chem..

[15] Ambrosiadis I., Theodorakakos N., Georgakis S., Lekas S. (1994). Influence of thawing methods on the quality of frozen meat and drip loss. Fleishwirtschaft.

[16] Hong G.-P., Park S.-H., Kim J.-Y., Lee C.-H., Lee S., Min S.-G. (2005). The effect of thawing rate on the physiochemical properties of frozen ostrich meat. Food Sci. Biotechnol..

[17] Bevilacqua A., Zartzky N.E., Calvelo A. (1979). Histological measurements of ice in frozen beef. J. Food Technol..

[18] SAS (2000). SAS/STAT Users Guide.

[19] Hofbauer P., Smulders J.M. (2011). A summary of methods to assess major physical-chemical and sensory quality traits of fresh (whole tissue) meat. Game Meat Hygiene in Focus.

[20] Gonzalez-Sanguinetti S., Aňon M.C., Calvelo A. (1985). Effect of thawing rate on the exudate production of frozen beef. J. Food Sci..

[21] Ngapo T.M., Babare I.H., Reynolds J., Mawson R.F. (1999). Freezing and thawing rate effects on drip loss from samples of pork. Meat Sci..

[22] Wagner J.R., Aňon M.C. (1985). Effect of freezing rate on the denaturation of myofibrillar proteins. Food Sci. Technol..

[23] Huff-Lonergan E., Lonergan S.M. (2005). Mechanisms of water-holding capacity of meat: The role of post mortem biochemical and structural changes. Meat Sci..

[24] Doherty A.M., Sheridan J.J., Allen P., McDowell D.A., Blair I.S. (1996). Physical characteristics of lamb primals packaged under vacuum or modified atmospheres. Meat Sci..

[25] Otremba M.M., Dikeman M.E., Boyle E.A.E. (1999). Refrigerated shelf-life of vacuum-packaged, previously frozen ostrich meat. Meat Sci..

[26] Warriss P.D. (2010). Meat hygiene, spoilage and preservation. Meat Science: An Introductory Text.

[27] Hoffman L.C., Botha S.S.C., Britz T.J. (2007). Muscle pH and temperature changes in hot- and cold-deboned ostrich (*Struthio camelus* var. *domesticus*) *muscularis gastrocnemius*, *pars interna* and *muscularis iliofibularis* during the first 23 h post mortem. Meat Sci..

[28] Shange N., Gouws P.A., Hoffman L.C. (2019). Changes in pH, colour and the microbiology of black wildebeest (*Connochaetes gnou*) *longissimus thoracis et lumborum* (LTL) muscle with normal and high (DFD) muscle pH. Meat Sci..

[29] Neethling N.E., Suman S.P., Sigge G.O., Hoffman L.C., Hunt M.C. (2017). Exogenous and endogenous factors influencing color of fresh meat from ungulates. Meat Muscle Biol..

[30] Lanari M.C., Bevilacqua A.E., Zaritzky N.E. (1989). Pigment modifications during freezing and frozen storage of packaged beef. J. Food Proc. Eng..

[31] Farouke M.M., Wieliczko K.J., Merts I. (2003). Ultra-fast freezing and low storage temperatures are not necessary to maintain the functional properties of manufacturing beef. Meat Sci..

[32] Farouke M.M., Swan J.E. (1998). Effect of muscle condition before freezing and simulated chemical changes during frozen storage on the pH and colour of beef. Meat Sci..

[33] Tomás M.C., Anón M.C. (1990). Study of the influence of freezing rate on lipid oxidation in fish (salmon) and chicken breast muscles. Int. J. Food Sci. Technol..

[34] Hansen E., Juncher D., Henckel P., Karlsson A., Bertelsen G., Skibsted L.H. (2004). Oxidative stability of chilled pork chops following long term frozen storage. Meat Sci..

[35] Benjakul S., Bauer F. (2001). Biochemical and physicochemical changes in catfish (*Silurus glanis linne*) muscle as influenced by different freeze/thaw cycles. Food Chem..

[36] Shanks B.C., Wulf D.M., Maddock R.J. (2002). Technical note: The effect of freezing on Warner-Bratzler shear force values of beef *longissimus* steaks across several post mortem aging periods. J. Anim. Sci..

[37] Petrović L., Grujić R., Petrović M. (1993). Definition of the optimal freezing rate - 2. Investigation of the physico-chemical properties of beef *M. longissimus dorsi* frozen at different freezing rates. Meat Sci..

